# Need for restoration of cortisol serum levels for successful antimicrobial therapy in experimental sepsis

**DOI:** 10.1186/cc12008

**Published:** 2013-03-19

**Authors:** T Doulias, A Pistiki, P Christopoulos, V Papaziogas, E Giamarellos-Bourboulis, I Koutelidakis

**Affiliations:** 1 University of Thessaloniki, Medical School, Thessaloniki, Greece; 2 University of Athens, Medical School, Athens, Greece

## Introduction

It is postulated that clinical benefit of low-dose hydrocortisone in septic shock is related to reversal of relative adrenal insufficiency [1]. This was proved in an animal model of sepsis.

## Methods

Sixty-nine Wistar male rats were assigned to the following groups: A, sham-operation; B, sepsis; C, bilateral adrenalectomy and sepsis; D, bilateral adrenalectomy, sepsis and hydrocortisone treatment; E, bilateral adrenalectomy, sepsis and ertapenem treatment; and F, bilateral adrenalectomy, sepsis, hydrocortisone and ertapenem treatment. Sepsis was induced by the i.p. infusion of 1×10^6 ^cfu/ml of *Escherichia coli *after adrenalectomy. Hydrocortisone 10 mg/kg was infused i.v. bid starting 1 hour after bacterial challenge. Ertapenem 5 mg/kg was infused i.v. once daily starting 1 hour after bacterial challenge. Survival was recorded. In a separate set of experiments in 18 rats, animal sacrifice was performed to measure the free cortisol concentration.

## Results

**Figure 1:**
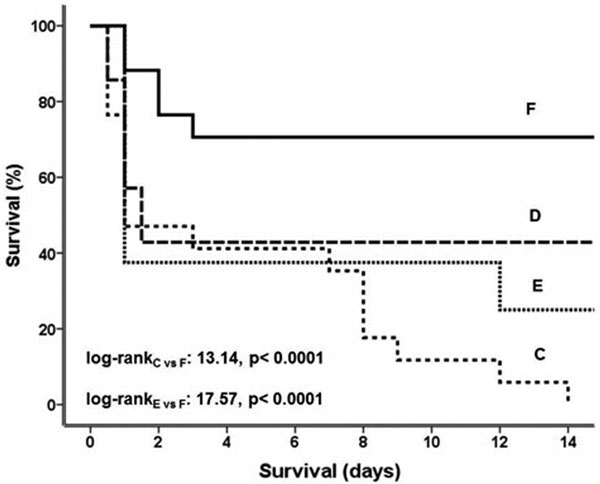
**Survival of rats**.

Survival is shown in Figure 1. Experiments in each animal were starting at 7:00 am. At 8:00 am, respective mean free cortisol of groups A, C and D was 1.81, 0.55 and 2.05 μg/dl; at 1:00 pm they were 0.92, 0.47 and 1.40 μg/dl.

## Conclusion

Even when effective antimicrobial treatment is administered, administration of hydrocortisone at a regimen restoring normal secretion is mandatory for survival.
